# Factors associated with COVID-19 stigma during the onset of the global pandemic in India: A cross-sectional study

**DOI:** 10.3389/fpubh.2022.992046

**Published:** 2022-10-14

**Authors:** Tulsi Adhikari, Sumit Aggarwal, Saritha Nair, Aparna Joshi, Vishal Diwan, A. Stephen, K. Rekha Devi, Bijaya Kumar Mishra, Girijesh Kumar Yadav, Sampada Dipak Bangar, Damodar Sahu, Jeetendra Yadav, Senthanro Ovung, Bal Kishan Gulati, Saurabh Sharma, Charan Singh, Chetna Duggal, Moina Sharma, Dhammasagar Ujagare, Sneha Padmakar Chinchore, Pricilla B. Rebecca, S. Rani, Pradeep Selvaraj, Gladston G. Xavier, Vanessa Peter, Basilea Watson, T. Kannan, K. S. Md. Asmathulla, Debdutta Bhattacharya, Jyotirmayee Turuk, Subrata Kumar Palo, Srikanta Kanungo, Ajit Kumar Behera, Ashok Kumar Pandey, Kamran Zaman, Brij Ranjan Misra, Niraj Kumar, Sthita Pragnya Behera, Rajeev Singh, Kanwar Narain, Rajni Kant, Seema Sahay, Rajnarayan R. Tiwari, Beena Elizabeth Thomas, M. Vishnu Vardhana Rao

**Affiliations:** ^1^ICMR-National Institute of Medical Statistics, New Delhi, India; ^2^Division of Epidemiology and Communicable Diseases (ECD), Indian Council of Medical Research, New Delhi, India; ^3^School of Human Ecology, Tata Institute of Social Sciences, Mumbai, Maharashtra, India; ^4^Division of Environmental Monitoring and Exposure Assessment (Water and Soil), ICMR-National Institute for Research in Environmental Health, Bhopal, Madhya Pradesh, India; ^5^Department of Social and Behavioral Research, ICMR-National Institute for Research in Tuberculosis, Chennai, Tamil Nadu, India; ^6^Enteric Disease Division, ICMR-Regional Medical Research Center, NE Region, Dibrugarh, Assam, India; ^7^Medical Department, ICMR-Regional Medical Research Center, Bhubaneswar, Odisha, India; ^8^ICMR-Regional Medical Research Center, Gorakhpur, Uttar Pradesh, India; ^9^Division of Epidemiology and Biostatistics, ICMR-National AIDS Research Institute, Pune, Maharashtra, India; ^10^Department of Environmental Health and Epidemiology, ICMR-National Institute for Research in Environmental Health, Bhopal, Madhya Pradesh, India; ^11^Division of Social and Behavioral Research, ICMR-National AIDS Research Institute, Pune, Maharashtra, India; ^12^Office of District Non-Communicable Disease, Directorate of Public Health and Preventive Medicine, Chennai, Tamil Nadu, India; ^13^Department of Social Work, Loyola College, Chennai, Tamil Nadu, India; ^14^Information and Resource Center for the Deprived Urban Communities, Chennai, Tamil Nadu, India; ^15^Electronic Data Processing Unit (EDP), ICMR-National Institute for Research in Tuberculosis, Chennai, Tamil Nadu, India; ^16^Epidemiology Statistics Unit, ICMR-National Institute for Research in Tuberculosis, Chennai, Tamil Nadu, India; ^17^Integrated People Development Project, Krishnagiri, Tamil Nadu, India; ^18^Department of Microbiology, ICMR-Regional Medical Research Center, Bhubaneswar, Odisha, India; ^19^Department of Epidemiology, ICMR-Regional Medical Research Center, Bhubaneswar, Odisha, India; ^20^Clinical Department, ICMR-Regional Medical Research Center, Bhubaneswar, Odisha, India; ^21^ICMR-Regional Medical Research Center, NE Region, Dibrugarh, Assam, India; ^22^Research Management, Policy, Planning and Coordination Cell, Indian Council of Medical Research, New Delhi, India; ^23^ICMR-National Institute for Research in Environmental Health, Bhopal, Madhya Pradesh, India

**Keywords:** COVID-19, stigma, stigmatizing attitudes, first wave, India

## Abstract

**Objective:**

To assess factors associated with COVID-19 stigmatizing attitudes in the community and stigma experiences of COVID-19 recovered individuals during first wave of COVID-19 pandemic in India.

**Methods:**

A cross-sectional study was conducted in 18 districts located in 7 States in India during September 2020 to January 2021 among adults > 18 years of age selected through systematic random sampling. Data on socio demographic and COVID-19 knowledge were collected from 303 COVID-19 recovered and 1,976 non-COVID-19 infected individuals from community using a survey questionnaire. Stigma was assessed using COVID-19 Stigma Scale and Community COVID-19 Stigma Scale developed for the study. Informed consent was sought from the participants. Univariate and multivariate binary logistic regression analysis were conducted.

**Results:**

Half of the participants (51.3%) from the community reported prevalence of severe stigmatizing attitudes toward COVID-19 infected while 38.6% of COVID-19 recovered participants reported experiencing severe stigma. Participants from the community were more likely to report stigmatizing attitudes toward COVID-19 infected if they were residents of high prevalent COVID-19 zone (AOR: 1.5; CI: 1.2–1.9), staying in rural areas (AOR: 1.5; CI:1.1–1.9), belonged to the age group of 18–30 years (AOR: 1.6; CI 1.2–2.0), were male (AOR: 1.6; CI: 1.3–1.9), illiterate (AOR: 2.7; CI: 1.8–4.2), or living in Maharashtra (AOR: 7.4; CI: 4.8–11.3). COVID-19 recovered participants had higher odds of experiencing stigma if they had poor knowledge about COVID-19 transmission (AOR: 2.8; CI: 1.3–6.3), were staying for 6–15 years (AOR: 3.24; CI: 1.1–9.4) in the current place of residence or belonged to Delhi (AOR: 5.3; CI: 1.04–26.7).

**Conclusion:**

Findings indicated presence of stigmatizing attitudes in the community as well as experienced stigma among COVID-19 recovered across selected study sites in India during the first wave of COVID-19 pandemic. Study recommends timely dissemination of factual information to populations vulnerable to misinformation and psychosocial interventions for individuals affected by stigma.

## Introduction

The outbreak of the novel Coronavirus Disease in 2019 (COVID-19) and public health preventive measures to contain the spread of the virus led to worry, uncertainty and fear among people (1). Further, lack of reliable information about the virus transmission and prevention, and apprehension about contracting it during the initial periods of the outbreak resulted in stigma and discrimination against people infected with or vulnerable to COVID-19 (2–4). Stigma is a social dynamic characterized by negative attitudes and exclusion of those who are perceived to be potential carriers of the disease (5). Stigmatization can increase unfavorable consequences of disease in multiple ways which could pose a challenge to the path of recovery. Literature review on experiences of people with Tuberculosis (TB), Human Immunodeficiency Virus (HIV) and Severe Acute Respiratory Syndrome (SARS) reported delay in testing and diagnosis, and non-adherence to or non-completion of treatment due to stigma or fear of stigma that led to increased disease transmission and impeded disease control (6–8).

Across the globe, several instances of COVID-19 stigma were reported among patients (and their families), persons suspected of having the infection, belonging to certain religious groups or geographical areas, people returning from overseas, healthcare workers, and migrant workers (2–5). A recent systematic review estimated prevalence of COVID-19 stigma (enacted stigma and perceived public stigma) as 35% [95% CI: 26–44%] (9) among affected individuals. People from low- and middle-income countries or with lower education were more vulnerable to stigma. In some countries, COVID-19 survivors continued to experience stigma even after the outbreak was well-contained (10).

Stigmatizing acts included social exclusion, stereotyping, insults, blame or threat, verbal abuse or gossip, physical abuse, denial of housing, and essential healthcare service including medicine, dismissal from job, and refusal from stores and restaurants during the pandemic (11–15). Being a part of a particular race, occupation, religious identity and social minority (migrants), illiteracy, poor knowledge, and lower income were reported to be some factors associated with COVID-19 stigma (5, 11).

Studies from India have documented stigma experienced by COVID-19 infected individual or those at risk; however, to our knowledge few have reported about the stigmatizing attitudes prevalent among those non-infected individuals and the factors associated with it, and about stigma experienced by those who were affected by COVID-19 or perceived to be affected by the same. Although, with greater understanding of COVID-19, its transmission pathways, treatment options, and better preventive measures including vaccination, there is a considerable decrease in stigma (16), instances of discrimination continue to exist in certain communities and groups. Hence, it is pertinent to understand the factors associated with stigma which will in turn inform strategies for mitigation. In this regard, a multi-centric study was conducted during the first wave of the COVID-19 pandemic in India to understand COVID-19 knowledge, risk perception, preventive measures and stigma so as to suggest appropriate mitigation strategies for minimizing stigma related to COVID-19. The study aimed to assess stigmatizing attitudes toward COVID-19 infected; stigma experienced by COVID-19 recovered individuals and factors associated with stigmatizing attitudes and experienced stigma.

## Methodology

### Study design

A cross-sectional national level study was conducted in 18 districts (administrative divisions) located in 7 States (Delhi, Uttar Pradesh, Madhya Pradesh, Odisha, Assam, Tamil Nadu, and Maharashtra) representing Central, East, North, North East, South, and West zones in India during the pandemic outbreak in the country (September 2020 to January 2021). The Ministry of Health and Family Welfare (MoHFW), India order dated 30/04/2020 number 28015/19/2020-EMR was used to select the states and districts according to the prevalence of COVID-19 epidemic (red zone indicating high prevalence and green zone indicating no cases until then). Out of the 18 districts, 12 belonged to the red zone and 6 to the green zone. For the study purpose, COVID-19 recovered individuals were defined as persons who were COVID-19 positive and had recovered and completed their isolation/hospitalization period, while, non-COVID-19 participants from the community were defined as persons who had not been infected with COVID-19 till the time of the survey.

Participants for the study included adults above the age of 18 years. Assuming prevalence of 30% stigmatizing attitudes in the community with 10% margin of error, 5% level of significance and design effect of 1.5, the sample size calculated was 1,800 for non-COVID-19 respondents. For COVID-19 recovered respondents, assuming prevalence of 70% experienced stigma, 16% margin of error, 5% level of significance and with design effect 1.5, the sample size calculated was 302. The required sample size for both non COVID-19 participants and COVID-19 recovered was equally distributed among 18 districts.

### Tools

A Survey questionnaire was designed to elicit information on socio-demographic characteristics, COVID-19 related knowledge (cause, transmission mode, symptoms and preventive measures), risk perception for the family and self, place of quarantine (for COVID-19 recovered participants) and COVID-19 stigma. Given the absence of standardized scales for measuring COVID-19 related stigma, the research team referred to the existing established framework (17) and researched scales for measuring HIV related stigma (18, 19). The HIV stigma frameworks (18, 19), for example, comments on the interaction between the individual and societal level factors in triggering stigma, the power differentials between those who are infected and non-infected, and also the differing mechanisms of stigma (manifested through enacted, anticipated and internalized stigma for those who are infected and through the prejudiced attitudes, discriminatory behaviors for those who are not infected). Hence, experienced stigma among COVID-19 recovered and prevailing stigmatizing attitudes displayed by the non-infected community members were assessed using two different scales (COVID-19 Stigma Scale and Community COVID-19 Stigma Scale) in the present study. Drawing from the HIV stigma framework, the Community COVID-19 stigma scale, comprising 6 statements, assessed prejudice, labeling, and discrimination by the non-infected community members. On the other hand, drawing from the same framework and the HIV stigma scale, COVID-19 stigma scale, comprising 13 statements, measured personalized stigma (perceived negative results of others knowing about the person's disease status), disclosure concerns (hiding information or worrying about breach of information) and concerns with public attitudes toward COVID-19 disease (harmful consequences of public attitudes). Details of scale development and pilot testing are available elsewhere (20). Survey questionnaires were translated to local languages (Hindi, Oriya, Tamil, Marathi, and Assamese). Due to restrictions imposed on conducting face-to-face data collection during COVID-19 pandemic, telephonic surveys were conducted by trained investigators across the study sites. Data collected was entered into the Census and Survey Processing System (CSPro) and later transferred to SPSS for analysis.

### Participants

*Community (non-COVID-19) participants*: The contact tracing list of COVID-19 infected persons above 18 years of age maintained by the health department as well as beneficiary data available with community-based organizations from the respective study areas were used to prepare a heterogenous and representative frame of non-COVID-19 participants from the community. The participants were selected from this frame using systematic random sampling Information was elicited from a total of 1,976 participants who had not been infected with COVID-19 till the date of the survey administration.

*COVID-19 recovered participants*: The sampling frame was prepared using the list of COVID-19 recovered individuals, as provided by the district health officials or the institutes conducting COVID-19 diagnosis between May and July 2020. A systematic random sampling procedure was used separately for the selection of the female and male participants. A total of 303 participants were included in the study.

The selected participants from both the groups were informed about the study and consent was sought orally over telephone; those who consented were included in the study. Total response rate ranged from 11.5% in Tamil Nadu to 43% in Odisha with an overall response rate of 22%. The success rates of contacting participants depended on the completeness and accuracy in obtaining telephone numbers of the selected participant in the sample frame and this may have induced bias. Few of the challenges reported by the sites in conducting telephonic surveys included: wrong numbers, discontinued numbers, participants not interested in the study, phone number in the name of another family member and network coverage issues. Persons not owning a mobile such as those from low-income communities, rural areas may have got excluded and also, since the participants were selected from lists available with health departments or community-based organizations, the population dynamics may have been different than the general population. However, given the urgency of conducting the study for providing information for mitigating stigma, telephonic surveys were the only possibility.

For ensuring the quality of data across the study sites, a manual was prepared to guide the investigators in collection of accurate information and training was conducted on best practices for telephonic data collection and recording information. Supportive supervision was provided, and data collected from each site was verified. Skipping and range checks were incorporated in the data entry forms and 10% post-entry check from the hard copies of the data were carried out. Data validation using frequency distributions at the time of data analysis was conducted.

### Ethical considerations

The study proposals and data collection tools were reviewed and approved by the Indian Council of Medical Research (ICMR)-National Task Force for Operations Research for COVID-19, ICMR-Central Ethics Committee for Human Research for COVID-19 (File No. NCDIR/BEU/ICMR-CECHR/75/2020, reference number: CECHR 015/2020 dated 10^th^ June, 2020) and the Ethical Review Committees of all the institutes participating in the study. Scientific robustness and accountability were audited by the ICMR Institute's Annual Scientific Advisory Committees (SAC). Participant Information Sheet (PIS) and Informed Consent (IC), translated to local languages, were read out to the participant over the phone and shared where ever possible through email or whatsapp. Consent was sought from the participants and recorded by the investigators from the respective sites.

#### Data sharing

Data was available with the investigators. Necessary government approvals were sought for sharing data.

#### Patient and public involvement statement

Patients or public were not involved in the conduct of research.

#### Transparency statement

The lead authors affirm that the manuscript is an honest, accurate, and transparent account of the study being reported, that no important aspects of the study have been omitted, and that there are no discrepancies from the study as originally planned.

#### Role of the funding source

The study was funded by the ICMR and had no role in the study design, collection of data, analysis and interpretation of data, writing of the report, and in the decision to submit the article for publication. The authors also confirm the independence of all researchers from funders and that all authors, external and internal, had full access to all of the data (including statistical reports and tables) in the study. The authors also take responsibility for the integrity of the data and the accuracy of the data analysis.

### Measurements

Independent variables were chosen as per literature review (5, 11–15) and expert advice. These included State, zone (red, green), socio demographic profile of the participant, place of quarantine (home/institution), any family member (s) with COVID-19 positive (yes, no), knowledge of cause (yes, no), transmission (yes, no), symptoms (>4, at least 4) and preventive measures (>3, at least 3), and risk perception of COVID-19 (unlikely, neutral, likely).

### Dependent variables

#### COVID-19 stigmatizing attitudes (outcome indicator)

Community COVID-19 Stigma Scale consisting of 6 statements assessed the stigmatizing attitudes of the community participants. Each statement was rated on a 3-point scale ranging from 0 = disagree to 2 = agree with higher scores indicating higher stigma attitudes. All the 6 statements were in the same direction. In the case of Community-19 stigma Scale, total score ranged from 0–12. The reliability of the scale was 0.60 and the median score was 6 (Table 1). In the absence of valid cut off points, it is generally advisable to use tertiles or quartiles to categorize scale score (21, 22). In the study, based on the sample size, tertile distribution was considered appropriate to categorize the stigma scores. The tertile distribution stigma score for community participants were 4 and 6 and were used as cut off points. Based on the categorization of the participants as per tertile distribution stigma scores ranged from no/mild stigma (<4), moderate stigma (4–5) and severe stigma (6+), and 51.3% of participants from the community displayed severe stigmatizing attitudes toward COVID-19 patients. A separate binary stigma variable was developed recoding the tertile stigma score of <4 as 0 and else 1 for binary logic regression analysis.

**Table 1:** Community COVID-19 stigma scale and COVID-19 stigma scale.

	**Community COVID-19 stigma scale**	**COVID-19 stigma scale**
	**(*N =* 1,976)**	**(*N =* 303)**
Reliability (Cronbach Alpha)	0.60	0.855
Mean (SD), Median	5.4 (3.09), 6	7.8 (6.9), 6
Range	0–12	0–26
**Tertiles**		
33	< 4 (T1): 33% of the participants had score < 4	< 2 (T1): 33% of the participants had score < 2
66	< 6 (T2) 66% of the participants had score < 6	< 10 (T2) 66% of the participants had score < 10
Category for stigma	0–3: No stigma/ mild; 4–5: moderate and >5 severe stigmatizing attitudes	0–1: No / mild; 2–9: moderate and >9 severe stigma
No/mild	25.3%	19.5%
Moderate stigma	23.4%	41.9%
Severe	51.3%	38.6%

#### Experienced COVID-19 stigma (outcome indicator)

A total of 13 statements assessed the stigma experiences of COVID-19 recovered participants. Each statement in the scale was rated on a 3-point scale ranging from 0 = disagree to 2 = agree with higher scores indicating higher experienced stigma. All 13 statements were framed in the same direction, to sustain logical interpretation and reduce the need for reversed responses. Total score ranged from 0–26. This composite stigma score was categorized based on tertiles, mild (less than 1st tertile stigma score), moderate (between 1^st^ and 2^nd^ tertile stigma score) and severe stigma (≥ 2^nd^ tertile stigma scores). The tertile stigma score for COVID-19 recovered participants were 2 and 10 and were used as cut off points. The reliability of the scale was 0.85 and the median score was 6 (Table 1). Based on the categorization of the participants as per tertile distribution stigma scores no/mild stigma (<2), moderate stigma (2–9) and severe stigma (10+), 38% of the participants reported experiencing severe stigma. A separate binary stigma variable was developed recoding the tertile stigma score of <1 as 0 and else 1. This recoded variable was used for binary logic regression analysis.

### Statistical analysis

To study the bivariate association between the outcome variable and the background characteristics and covariates, test of significance with cross tabs, chi-square test with *p*-value were conducted. The multivariate binary logistics regression analysis was conducted between the recoded outcome variable (no or mild stigma as 0 and else 1) and the variables which were significantly associated with stigma in the bivariate analysis. The multivariate binary logistic regression gave the adjusted Odds ratio, *p*-value and Confidence Interval of the adjusted ORs, adjusting for the confounding effect of all the other covariates.

## Results

### Profile of participants

**Figure 1:**
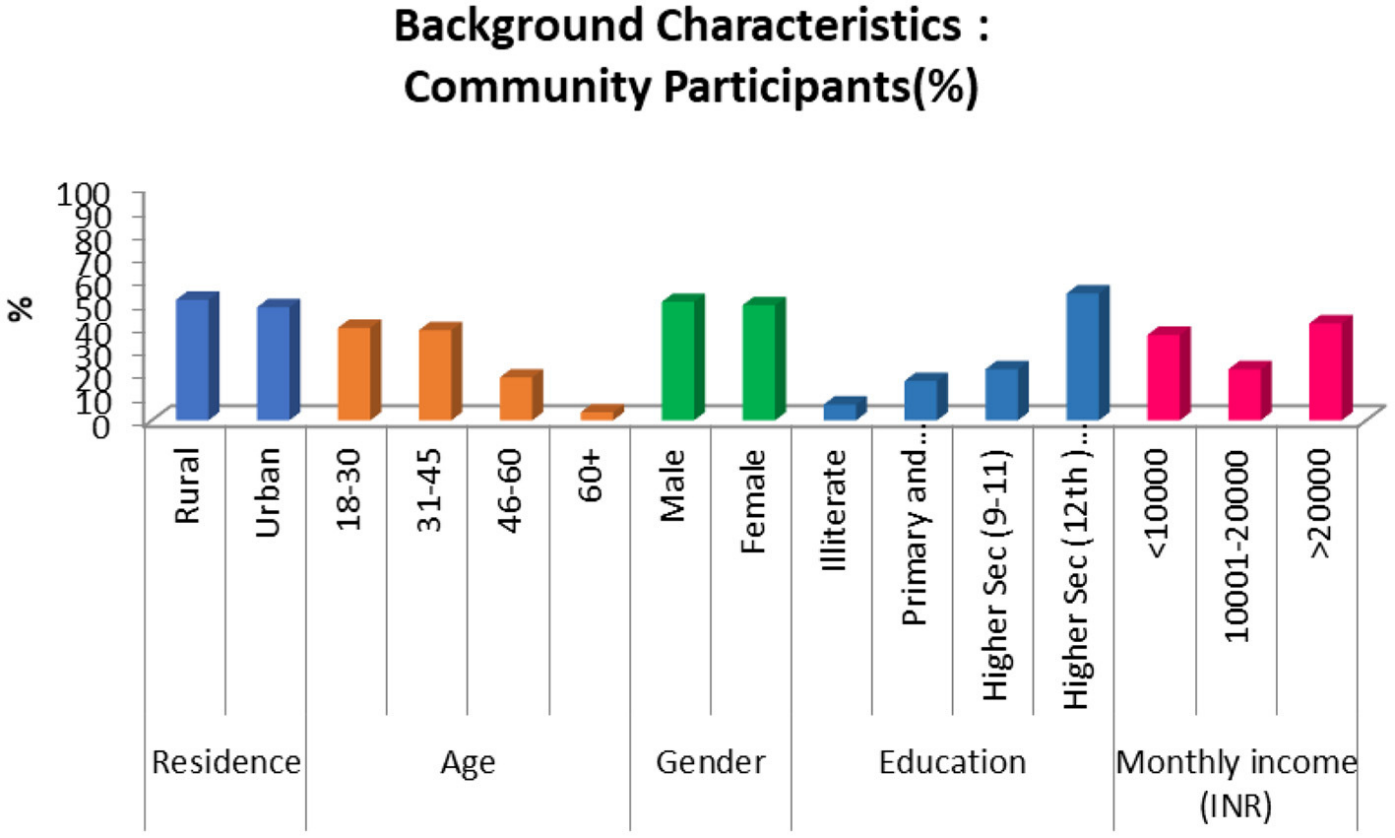
Socio-demographic characteristics of community participants.

The mean age of the community participants (*n* = 1,976) was 36 years, 71.8% were married, 54.3% had higher secondary and above education, and 51% of the participants resided in urban areas (Figure 1). Nearly three-fifths perceived no risk of getting infected with COVID-19.

**Figure 2:**
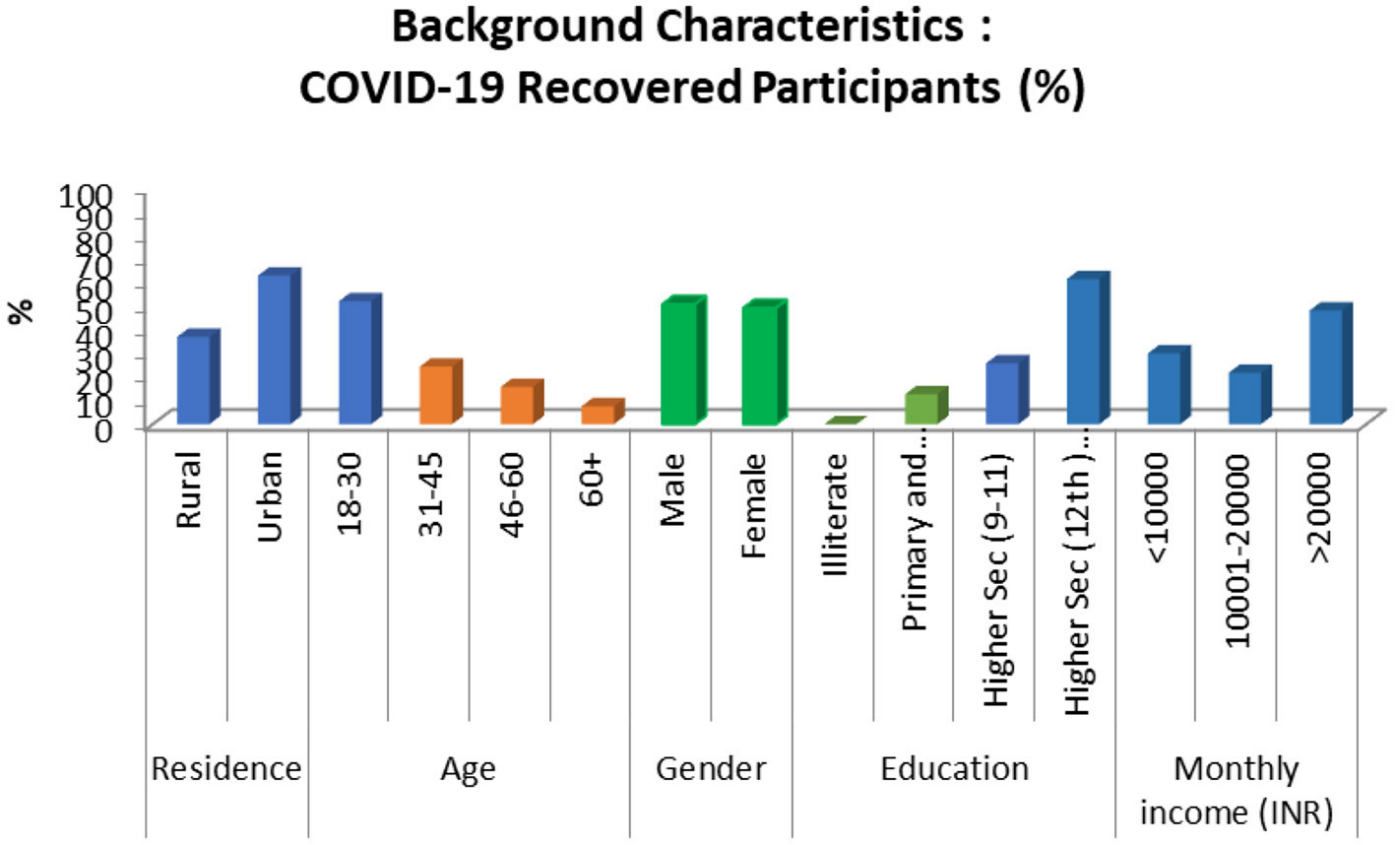
Socio-demographic characteristics of COVID-19 recovered participants.

The mean age of the COVID-19 recovered participants (*n* = 303) was 38 years, 69% were married, 61.5% had higher secondary and above education, and 63% were residing in urban areas (Figure 2). Many (83%) participants reported of institutional quarantine during the time they were COVID-19 positive.

Majority of the participants from the community reported knowledge about the cause (66.0%), modes of transmission (69.0%), symptoms (54.0%) and preventive measures of COVID-19 (75.0%). Similar results were observed for the COVID-19 recovered participants.

### Factors associated with COVID-19 stigmatizing attitudes in the community and stigma experiences

Table 2 illustrates the percentage distribution of COVID-19 stigmatizing attitudes by selected socio demographic and COVID-19 related variables. Majority of community participants from Odisha (92%) and Maharashtra (90%) reported of moderate and severe stigmatizing attitudes. Participants from Assam reported the lowest (53%) stigmatizing attitudes (Table 2). Fifty-four percent of participants from rural as compared to 48% from urban area reported stigmatizing attitudes. Fifty percent of males compared to 47% of females reported of severe stigmatizing attitudes. Participants in the age group of 18–30 and 45–60 years reported of severe stigmatizing (56.1 and 52.5 %, respectively) attitudes. Community participants with a COVID-19 positive family member had less stigmatizing attitudes (67.8%) than those without (76%). All the above differences were statistically significant.

**Table 2:** Stigmatizing attitude among non-COVID-19 community participants and stigma experienced among COVID-19 recovered participants by selected variables (socio demographic, COVID-19 knowledge and risk perception) (bivariate analysis).

	**COVID-19 stigmatizing attitudes**					**Experienced COVID-19 stigma**				
	**No/Mild**	**Moderate**	**Severe**	**n**	***p*-value**	**No/Mild**	**Moderate**	**Severe**	***n***	***p*-value**
**Total**	25.3	23.4	51.3	1,976		19.5	41.9	38.6	303	
**Age group (years)**	
18–29	22.6	21.2	56.1	782	0.008	24.1	41.7	34.3	108	0.586
30–44	27.3	26.6	46.1	763		14.9	40.4	44.7	94	
45–59	26.5	21	52.5	362		19.5	45.5	35.1	77	
≥60	27.5	24.6	47.8	69		16.7	37.5	45.8	24	
**Sex**	
Male	21.6	23.1	55.4	1,002	< 0.001	16.2	45.5	38.3	154	0.268
Female	29.2	23.7	47.1	974		22.8	38.3	38.9	149	
**Completed years of schooling**										
Illiterate	23.1	18.7	58.2	134	< 0.001	20	50	30	20	0.259
1–10 std	21.8	22.5	55.7	743		18.3	34.6	47.1	104	
11 and above	27.9	24.6	47.5	1,099		20.1	45.3	34.6	179	
**Occupation**	
Govt. employees	26.8	23.7	49.4	257	< 0.001	25.5	50.9	23.6	55	0.12
Pvt. Employees	25.3	30.2	44.5	391		22.5	35.2	42.3	71	
Skilled/unskilled labor/Self employed	20.5	22.0	57.5	610		11.1	44.4	44.4	18	
Others	28.8	20.8	50.4	718		19.1	44.3	36.5	115	
**Income in Indian rupees**										
< 10,000	24.6	21	54.4	723	0.139	19.8	38.5	41.8	91	0.462
10,001–20,000	23.6	24.5	52	433		22.7	34.8	42.4	66	
>20,000	26.8	24.9	48.3	820		17.8	47.3	34.9	146	
**Marital status**										
Never married	21.1	23.3	55.7	494	0.081	23.1	39.7	37.2	78	0.699
Currently married	26.6	23.7	49.7	1,420		18.7	41.6	39.7	209	
Separated	29	17.7	53.2	62		12.5	56.2	31.2	16	
**Religion**										
Hindu	25.5	23.3	51.2	1,729	0.844	20	39.2	40.8	255	0.152
Muslim	23.3	21.9	54.8	146		23.1	50	26.9	26	
Others	24.8	26.7	48.5	101		9.1	63.6	27.3	22	
**State**										
Madhya Pradesh	36.8	25.9	37.4	340	< 0.001	16.1	39.3	44.6	56	< 0.001
Odisha	7.7	18.1	74.2	326		25	18.8	56.2	48	
Delhi	31.8	36.4	31.8	110		9.5	42.9	47.6	21	
Uttar Pradesh	24.8	22	53.2	218		31.2	40.6	28.1	32	
Assam	47.1	24.2	28.7	327		31.2	47.9	20.8	48	
Tamil Nadu	22.4	26.6	51.1	331		8.3	58.3	33.3	48	
Maharashtra	10.2	18.5	71.3	324		14	46	40	50	
**COVID-19 zone**										
Red	27.6	23.4	48.9	1,259	< 0.001	22.8	40.8	36.4	206	0.095
Green	21.2	26.6	55.5	717		12.4	44.3	43.3	97	
**Place of residence**										
Urban	25.2	26.6	48.2	956	< 0.001	17.8	45.5	36.6	191	0.236
Rural	25.4	20.4	54.2	1,020		22.3	35.7	42	112	
**Duration of residence in the place**	
< 5 years	21.5	20.8	57.8	303	< 0.001	32.7	38.2	29.1	55	0.015
6–15 Years	30.2	27.5	42.2	334		12	56	32	50	
>15 Years	24.9	22.9	52.1	1,339		17.7	39.4	42.9	198	
**Place of quarantine**	
Home	**Not applicable**					16	42	42	50	0.76
Institution						20.2	41.9	37.9	253	
**Family member infected with COVID-19**										
Yes	32.2	24.6	43.2	301	< 0.001	15.4	40.6	44.1	143	0.104
No	24.1	23.2	52.8	1,675		23.1	43.1	33.8	160	
**Knowledge of cause of COVID-19**										
Yes	25.8	24.2	50	1,306	0.25	20.3	40.6	39.1	202	0.778
No	24.3	21.8	53.9	670		17.8	44.6	37.6	101	
**Knowledge of COVID-19 transmission**										
No	24.7	22.2	53.1	599	0.561	10.2	50	39.8	88	0.024
Yes	25.6	23.9	50.5	1,377		23.3	38.6	38.1	215	
**Knowledge of symptoms** (COVID-19 recovered median = 3 symptoms; community participants median = 4 symptoms)
< 3 symptoms	26.6	22.1	51.3	903	0.342	15.1	48.8	36	86	0.253
Atleast 3 symptoms	24.2	24.4	51.4	1,073		21.2	39.2	39.6	217	
**Knowledge of preventive measures (median score** **=** **3)**										
< 3 preventive measures	26.9	25.1	48	487	0.251	11.8	43.4	44.7	76	0.131
Atleast 3 preventive	24.8	22.8	52.4	1,489		22	41.4	36.6	227	
**Risk perception**										
Unlikely	25	21.3	53.7	1,151	0.081	Not applicable				
Neutral	25.6	26.5	47.9	426		
Likely	25.8	26.1	48.1	399		

* Significant p value < 0.05.

** Significant p value < 0.001.

The multivariate binary logistic regression analysis for factors associated with stigmatizing attitudes, revealed that the inter-state differences were statistically significant (*p* < 0.001) with least stigmatizing attitudes reported in Assam (Table 3). Stigmatizing attitudes were significantly higher among the participants from Maharashtra (AOR = 7.3), and Odisha (AOR = 6.3). People living in red (high COVID- 19 prevalence) zones and rural areas had more stigmatizing attitudes with adjusted odds ratio of around 1.5. The difference between red and green (zero prevalence) zones were statistically significant. Individuals in the age group of 31–45 years had less stigmatizing attitudes as compared to the younger age group, i.e., 18–30 years (AOR = 1.6) or the older age group (AOR = 1.4), i.e., 46–60 years. Men had more stigmatizing attitudes (AOR = 1.6) toward COVID-19 patients. Illiterate participants had more stigmatizing attitudes than those with education higher than secondary level (AOR = 2.7).

**Table 3:** Factors associated with stigma experience among the COVID-19 recovered participants and stigmatizing attitude among non-COVID-19 community participants (multivariate analysis).

	**Non COVID-19 community participants (stigmatizing attitudes)**			**COVID-19 recovered participants (experienced stigma)**		
	**Sig**.	**Adj OR**	**95% CI**	**Sig**.	**AOR**	**95% CI**
**Age group (years)**
31–45 (Ref)	< 0.001			
18–30	0.0	1.558	1.229 1.975	
46–60	0.02	1.423	1.057 1.915	
60+	0.961	1.016	0.545 1.894	
**Sex**
(Ref - Female)				
Male	0	1.561	1.266 1.926	
**Education**
11^th^ and higher (Ref)				
Illiterate	0	2.734	1.761 4.246	
1–10 std	0	1.512	1.203 1.899	
**Knowledge about COVID-19 transmission**
Yes (Ref)				
No				0.011	2.829	1.267 6.319
**Place of residence**
Urban (ref)				
Rural	< 0.001	1.45	1.13 1.86	
**Duration of residence in the current place**
< 6 years (Ref)				0.054		
6 to 15 years				0.03	3.24	1.117 9.397
>15 years				0.051	2.089	0.998 4.375
**COVID-19 zone**
(Ref-Green)				
Red	< 0.001	1.492	1.148 1.940	
**State**
Assam (Ref)	< 0.001			0.058		
Madhya Pradesh	0.024	1.554	1.059 2.282	0.156	2.049	0.760 5.529
Odisha	0	6.314	4.232 9.419	0.73	1.18	0.460 3.025
Delhi	0.217	1.409	0.817 2.429	0.044	5.278	1.043 26.695
Uttar Pradesh	0	2.189	1.419 3.376	0.952	0.969	0.352 2.666
Tamil Nadu	0	3.184	2.153 4.709	0.026	4.009	1.177 13.667
Maharashtra	0	7.379	4.825 11.286	0.054	2.771	0.983 7.812

* Variables significant in bivariate analysis were considered for the multivariate analysis.

Significant differences were observed in stigma experiences of COVID-19 recovered participants based on the State to which they belonged. A little more than half (56%) of COVID-19 recovered participants from Odisha reported of experiencing severe stigma compared to 28% from Uttar Pradesh (Table 2). Stigma experiences were significantly different among residents who were staying at their current residential address for more than 5 years than those who were living at the current place for short duration of time. Experienced stigma was significantly higher among participants who did not know about the mode of transmission of COVID-19 infection.

Multivariate logistic regression model for factors associated with reporting of experienced stigma by COVID-19 recovered individuals is presented in Table 3. Experiences of COVID-19 stigma were statistically significant and more likely to be reported by COVID-19 recovered individuals who belonged to the state of Delhi (AOR = 5.28) and Tamil Nadu (AOR = 4.01). COVID-19 recovered individuals who were staying at the place of residence (district) for more than 6 years experienced more stigma as compared to those who were residing at the current place for <6 years (AOR>2). Individuals who had good knowledge about modes of transmission of COVID-19 were less likely to have experienced stigma as compared to those who did not have the knowledge (AOR = 2.83).

## Discussion

The widespread stigma associated with COVID-19 experienced by many and reported by media especially during the initial phases had devastating health consequences such as prompting people to hide the illness and preventing from seeking help and adopting healthy behaviors (1–3, 5). It also led to debilitating psychological and social consequences (12, 15, 23). To design targeted strategies for information dissemination, disease prevention, and stigma mitigation in India, a multi-centric study was undertaken during the onset of COVID-19 pandemic in India. The aim of the study was to understand COVID-19 stigmatizing attitudes in the community and stigma experienced by COVID-19 recovered individuals as well as factors associated with the same. Findings from this study document that nearly three-fourths of the study participants reported of stigmatizing attitudes and majority of the COVID-19 recovered participants had experienced some levels of stigma. Similar findings on stigma experiences of COVID-19 patients were reported from studies conducted in India and China (24, 25). Stigmatizing attitudes and discrimination toward COVID-19 patients were also observed among 60–80% of individuals from the general population in China and Jordan (26, 27). Higher levels in reporting of stigma, both experienced and stigmatizing attitudes, may be due to fear and paucity of knowledge on prevention or possible treatment options during the COVID-19 outbreak in India when the study was conducted. Fear of infection has been reported to be associated with heightened perceived stigma (28, 29). These results have implications for developing strategies in mitigating stigmatizing attitudes in the community and providing support to those who may experience stigma particularly during the initial phases of any infectious disease outbreak.

In this study, severity of stigma experienced by COVID-19 recovered individuals as well as prevailing stigmatizing attitudes in the community were associated with the state in which the participants resided at the time of the interview. Participants selected for the study belonged to districts located in States that had higher number of COVID-19 confirmed cases during the first wave and were declared red zones during the outbreak in the country (30). News reports had also highlighted the presence of COVID-19 stigma in these locations (31). Similar reports of increased COVID-19 stigma experiences were reported by individuals residing in highly affected countries or in hotspot zones (32–34). Also, residents living in geographical locations with the greatest number of cases reported higher levels of stigmatizing attitudes due to fear for potential infection (35, 36).

Good knowledge about COVID-19 was significantly associated with lesser stigma experiences. Having an appropriate knowledge on COVID-19 pandemic may have helped in judging misinformation and stereotypes (37) resulting in reduced stigma experiences. However, this is contrary to the study conducted by Saine and colleagues (38) that reported an increase in perceived stigma among patients who had hepatitis C virus (HCV)-related knowledge.

Stigmatizing attitudes were found more among the younger (18–30) and older (46–60) age groups of community participants. Older population had significantly higher stigmatizing attitudes toward COVID-19 infected due to higher perceived susceptibility and severity of COVID-19 (33, 39). Our study findings differ from other studies that reported lower stigmatizing attitudes among older (40) and younger adults (39, 41). Higher stigmatizing attitudes among younger population may have been due to their heightened exposure to misinformation which was widely circulated through social media groups. Male compared to female participants in the study had higher stigmatizing attitudes toward COVID-19 infected. The findings are consistent with previous studies on COVID-19 (33, 42, 43). Fear of increased risk of morbidity and mortality reported among men due to COVID-19 during the initial phases of pandemic may have resulted in higher stigmatizing attitudes among this group (44).

Our findings show that community participants with lower literacy levels were more likely to have stigmatizing attitudes toward COVID-19 infected. Education level of an individual could have a significant influence on their knowledge and thereby result in lesser stigmatizing attitudes (23, 45). Similar findings have been stated in several studies (13, 33, 40, 46–48), which reported that participants who had difficulties to find and understand information about COVID-19 were more likely to have stigmatizing attitudes toward people with the infection.

The present research not only corroborates media reports published during the onset of COVID- pandemic in India regarding stigma experienced by majority of COVID-19 infected individuals, but also provides supporting evidence for the presence of stigmatizing attitudes and factors associated with the same among non-COVID-19 infected individuals from the community Given the devastating health, social and psychological consequences of COVID-19 pandemic, our study findings call for timely deployment of anti-stigma programmes along with public health protective measures for mitigation of discriminatory attitudes and stigma experiences that may interfere with overall health and wellbeing and come in way of pandemic containment responses. For example, responding to initial media reports of COVID-19 stigma and its impact, the Ministry of Health and Family Welfare (MoHFW) in India released guidelines on do's and don'ts for mitigation of stigma. Likewise, other initiatives undertaken by the GoI (Government of India) included psycho-social toll-free helpline and the “Break the Stigma” campaign (49). Such steps not only eased the struggle of the COVID-19 affected individuals against stigma but also dealt with the infodemic of misinformation and rumors that played a crucial role in creating stigma.

In addition to dissemination of correct information as currently undertaken by the Government and other organizations engaged in infection prevention, the study also emphasizes the need to particularly focus on populations more vulnerable to misinformation. These include less educated, those living in high prevalence States, people living in rural areas or migrant workers. Since lack of proper knowledge and poor literacy resulting in fear are major factors associated with stigma, mass media, and social media outreach could be leveraged to disseminate updated, accurate and easily understandable information, dispel myths, fears and stigmatizing attitudes, and promote empathic behaviors toward those infected. Lastly, the study recommends the need for timely psychosocial interventions to alleviate negative impacts of stigma in individuals affected by COVID-19 and to provide necessary support.

## Strengths and limitations

Certain limitations may be considered while interpreting the results of the present study. Collection of sensitive information on stigma experiences and attitudes through surveys, in the absence of face to face methods of data collection may have induced biases. This also might have resulted in greater non-response rates. Since, findings are based on participants chosen from selected districts in India, the results although largely indicative of the COVID-19 stigma situation in India, may not be generalizable. A cross sectional study design may have posed challenges in assessing the factors associated with COVID-19 stigma as both stigma and its independent variables were examined at the same time. Use of robust methodology, triangulation of COVID−19 stigma from stigmatized and stigmatizers from major geographical zones affected by COVID-19 during the first wave in India are the strengths of the study.

## Conclusion

Study indicates the presence of COVID-19 stigma in the study population and emphasizes the need for timely interventions to mitigate stigma by increasing awareness and knowledge on COVID-19.

## Data availability statement

The datasets presented in this article are not readily available because data is available with the investigators. Necessary government approvals would have to be sought for sharing data. Requests to access the datasets should be directed to nairs@icmr.gov.in.

## Ethics statement

The studies involving human participants were reviewed and approved by ICMR-Central Ethics Committee for Human Research for COVID-19 (File No. NCDIR/BEU/ICMR-CECHR/75/2020, Reference Number: CECHR 015/2020 dated 10th June, 2020). Written informed consent was not provided because Participant Information Sheet (PIS) and Informed Consent (IC), translated to local languages, were read out to the participant over the phone and shared where ever possible through email or whatsapp messenger. Consent was sought from the participants and recorded by the investigators from the respective sites.

## Author contributions

SB, JY, BG, SSh, CS, CD, MS, DU, SPC, PR, SR, PS, GX, VP, BW, TK, KA, DB, JT, SKP, SK, AK, AP, KZ, BM, NK, SB, RS, KN, RK, and RT contributed by conducting critical review and editing. All authors contributed to the article and approved the submitted version.

## Funding

This study was financially supported by the Indian Council of Medical Research (ICMR), New Delhi (wide letter No. HIV/COVID-19/3/5/2020/ECD-II dated July 20th 2020).

## Conflict of interest

The authors declare that the research was conducted in the absence of any commercial or financial relationships that could be construed as a potential conflict of interest.

## Publisher's note

All claims expressed in this article are solely those of the authors and do not necessarily represent those of their affiliated organizations, or those of the publisher, the editors and the reviewers. Any product that may be evaluated in this article, or claim that may be made by its manufacturer, is not guaranteed or endorsed by the publisher.
