# Supplementary material for: Factors associated with COVID-19 stigma during the onset of the global pandemic in India: A cross-sectional study
Source: Front Public Health. 2022 Oct 14;10:992046. doi: 10.3389/fpubh.2022.992046 (PMC9615248; doi:10.3389/fpubh.2022.992046)
Supplement: Supplementary file 1 [file Data_Sheet_1.PDF]

Supplementary Files: **Interview Schedules**

COVID-19 RECOVERED RESPONDENTS-I

Factors Related to Covid-19 Stigma: A Mixed-Methods Study

SECTION 1: IDENTIFICATION

SECTION 2: SOCIOECONOMIC BACKGROUND

Q. No.	QUESTIONS AND FILTERS	CODING CATEGORIES		Skip To
Q201	Age (In completed years) 18-99			
Q202	Gender	Male	1	
		Female	2	
		Transgender	3	
Q203	Have you ever attended school	YES	1	If 2 go to Q205
		NO	2	
Q204	What is the highest grade you completed?<br>***FOR CODE REFER MANUAL***	Grade 1 to 20		If any grade 1 to 20 go to Q206
Q205	If never attended school	Can read and write only	1	
		Can read only	2	
		Don't know	3	
Q206	What is your occupation?	Government Employee	1	
		Private Employee	2	
		Skilled Labour	3	
		Unskilled Labour	4	
		Business/Self Employee	5	
		Unemployment	6	
		Housewife	7	
		Student	8	
		Others (please specify):	9	
Q207	What is your family's monthly income (INR)?	Up to 5000	1	
		5001-7500	2	
		7501-10000	3	
		10001-20000	4	
		20001-50000	5	
		50001-100000	6	
		Above 100000	7	
Q208	What is your current marital status?	Never married	1	
		Currently married	2	
		Separated	3	
		Divorced/ separated	4	
		Widower/Widow	5	
		Live-in relationship	6	
Q209	Which religion do you belong to?	Hindu	1	
		Muslim	2	
		Christian	3	
		Budhist	4	
		Others (please specify):	5	
Q210	Which caste/ tribe do you belong to?	Scheduled Caste	1	
		Scheduled Tribe	2	
		OBC	3	
		General	4	
		None of these	5	
Q211	Are you a usual resident of this place?	YES	1	

ICMR-National Institute of Medical Statistics, Delhi

		NO 2	
Q212	How long have you been residing in this place? (Completed years)	[ ][ ]	
Q213	Whether you or any member of your family have visited any place out of your current place of residence since 1st March 2020?	YES 1<br>NO 2	
Q214	Whether you or any member of your family has returned to your current place of residence after 1st March 2020?	YES 1<br>NO 2	
Q215	Where were you quarantined or isolated for COVID-19?	Home 1<br>Institution (hospital/hotel/any other) 2	

SECTION 3: UNDERSTANDING OF COVID-19

We would like to ask you some questions on COVID which refer to your knowledge on this virus before you were diagnosed.

Q. No.	QUESTIONS AND FILTERS	CODING CATEGORIES	Skip to
Q301	Before you were diagnosed with COVID-19, did you hear about COVID-19 or CORONA?	YES 1<br>NO 2	If 2 skip to Q 304
Q302	Through which sources did you come to know about COVID-19?<br><br>**[MULTIPLE RESPONSES]**	**Circle appropriate responses**<br>A Newspaper<br>B Magazine<br>C Radio<br>D Television<br>E Internet<br>F Social platforms (E.g. WhatsApp, WeChat, Fac Twitter, Instagram)<br>G Your doctor<br>H Your family or friends<br>I Other (please specify)	
Q303	Which one of the following sources of information do you trust the most?	Newspaper<br>Magazine<br>Radio<br>Television<br>Internet<br>Social platforms (E.g. WhatsApp, WeChat, Facebook, Twitter, Instagram)<br>Your doctor<br>Your family or friends<br>Other (please specify)	
Q304	COVID-19 is caused by:<br><br>**[MULTIPLE RESPONSES]**	**Circle appropriate responses**<br>A A germ<br>B Virus<br>C By Bat<br>D Eating sea food or any animal food/ meat products<br>E Other (please specify)	
Q305	COVID-19 transmitted through:<br><br>**[MULTIPLE RESPONSES]**	**Circle appropriate responses**<br>A Air (when a person coughs, sneezes or talks)<br>B Touching another person<br>C Unknowingly touching a surface/object which has the virus and then touching your eyes and nose<br>D Consumption of meat/chicken/sea food	

ICMR-National Institute of Medical Statistics, Delhi

Q.	Questions and filters		Coding categories	Code	Skip
		E	Visiting butcher shops and fish markets		
		F	Caused by contaminated water		
		G	Other (please specify)		
Q306	Symptoms of COVID19 are: **[MULTIPLE RESPONSES]**		**Circle appropriate responses**		
		A.	Fever		
		B.	Cough		
		C.	Difficulty breathing		
		D.	Nasal Congestion		
		E.	Running nose		
		F.	Sore Throat		
		G.	Loss of smell and Taste		
		H.	Fatigue		
		I.	Diarrhoea		
		J.	Constipation		
		K.	Rash		
		L.	Asymptomatic: No symptom		
		M.	Any Pain (head, body, muscle, chest, back)		
		N.	Other (please specify)		
Q307	Did you have any symptoms for COVID-19?		YES	1	If 2 go to Q309
			NO	2	
Q308	If yes, were you hospitalised?		YES	1	
			NO	2	
Q309	It is possible that the COVID positive individual can be completely be asymptomatic for some time		True	1	
			False	2	
			Don't know	3	
Q310	COVID-19 can be prevented through: **[MULTIPLE RESPONSES]**		**Circle appropriate responses**		
		A	Wearing a face mask		
		B	Washing hands frequently (With soap or hand saniti		
		C	Practising physical distancing		
		D	Avoiding contact with people who have sympto cough		
		E	Avoiding contact with people who have a travel his a foreign country		
		F	Avoiding crowded areas		
		G	Not visiting butcher shops and fish markets		
		H	Avoiding visits to a hospital or clinic		
		I	Avoiding public transportation		
		J	Being at home		
		I	Others (please specify)		
Q311	In your opinion, which of the 3 measures that people will adopt the most to prevent COVID transmission? **[MULTIPLE RESPONSES]**		**Circle appropriate responses**		
		A	Wearing a face mask		
		B	Washing hands frequently (With soap or hand sanitizer)		
		C	Practising physical distancing		
		D	Avoiding contact with people who have symptoms of cough		
		E	Avoiding contact with people who have a travel history to a foreign country		
		F	Avoiding crowded areas		
		G	Not visiting butcher shops and fish markets		
		H	Avoiding visits to a hospital or clinic		
		I	Avoiding public transportation		
		J	Being at home		
		H	Others (please specify)		

SECTION 4. RISK PERCEPTION

Q.	QUESTIONS AND FILTERS	CODING CATEGORIES	Skip

ICMR-National Institute of Medical Statistics, Delhi

No.					to
Q401	According to you, who are at the risk of getting COVID-19?	**Circle appropriate responses**			
	**[MULTIPLE RESPONSES]**	A	People who have a travel history from a foreign count		
		B	Young people		
		C	Children		
		D	Elderly		
		E	People living in crowded areas		
		F	People living in cities		
		G	People living in rural areas		
		H	Women		
		I	Men		
		J	People with any comorbidity		
		K	Other (please specify)		
Q402	According to you, who could spread COVID-19?	**Circle appropriate responses**			
	**[MULTIPLE RESPONSES]**	A	Doctors/health care workers		
		B	Police personnel		
		C	People who have a travel history from a foreign count		
		D	Young people		
		E	Children		
		F	Elderly		
		G	People living in crowded areas		
		H	People living in cities		
		I	People living in rural areas		
		J	Women		
		K	Men		
		L	People who do not practice social distancing		
		M	People who do not wear masks		
		N	Vendors (Vegetable, milk etc)		
		O	Other (please specify)		
Q403	Please elaborate the reasons why you think you might have become infected with COVID-19?	**Circle appropriate responses**			
	**[MULTPLE RESPONSES]**	A.	Come in close contact with COVID positive person		
		B.	Occupation		
		C.	Travel history from a foreign country		
		D.	Travel history from a place (in India) that has many COVID-19 people		
		E.	Elderly		
		F.	Prior illness (BP/Diabetes/TB/Cancer/ any other)		
		G.	Live in a crowded area		
		H.	Live in a place where infections are increasing		
		I.	Many people have come have come from cities to the rural area		
		J.	Pregnant		
		K.	Lactating mother		
		L.	Travel by public transportation		
		M.	Any other response (please specify)		
Q404	Did any of your family members who are staying with you test positive for COVID-19?		YES	1	If 1 skip to Q501
			NO	2	
Q405	How likely do you think your family members might become infected with COVID-19?		Very Unlikely	1	If 1 or 2 then go to Q407
			Unlikely	2	
			Neutral	3	
			Likely	4	
			Very likely	5	
Q406	Please elaborate the reasons why you think your family might	**Circle appropriate responses**			If A to K skip
		1.	Have come in close contact with COVID positive		

ICMR-National Institute of Medical Statistics, Delhi

	become infected with COVID-19?<br><br>**[MULTPLE RESPONSES]**		person	to Q501
		2.	Occupation that requires close interaction with COVID-19 positive people	
		3.	Travel history from a foreign country	
		4.	Travel history from a place (in India) that has many COVID-19 people	
		5.	Are above 60 years of age	
		6.	Have prior illness (BP/Diabetes/TB/Cancer/ any other)	
		7.	Live in a crowded area	
		8.	Live in a place where infections are increasing	
		9.	Many people have come have come from cities to the rural areas where they stay	
		10.	Travel by public transportation	
		K	Any other response (please specify)	
Q407	Please elaborate the reasons why you think/thought your family might not become infected with COVID-19?<br><br>**[MULTPLE RESPONSES]**	**Circle appropriate responses**		
		A.	Have not come in close contact with COVID positi person	
		B.	Always adhere to preventive measures	
		C.	Have no travel history from a foreign country	
		D.	Have no travel history from a place (in India) that h many COVID-19 people	
		E.	All are young	
		F.	Have no prior illness	
		G.	Do not live in a crowded area	
		H.	Live in a place where there are no infections	
		I.	Do not take by public transportation	
		J.	any other response (please specify)	

SECTION 5. COVID-19 STIGMA

READ THE FOLLOWING STATEMENTS CAREFULLY AND TICK ONE OPTION THAT APPLIES TO THE RESPONDENT.

We would like to understand your experiences with COVID-19. This understanding would help us provide information to help others diagnosed with COVID-19 to have better experiences as this is a new pandemic and there are still problems that need to be addressed. We will read some statements and we would like you to respond if you agree/ disagree or if you cannot say. I will be happy to repeat the question if I am not clear.

Sl. No.	STATEMENT	CODING CATEGORIES		Skip to
Q501	People I cared stopped calling or interacting after learning that I was infected with COVD-19.	Agree	2	
		Disagree	0	
		Can't Say	1	
Q502	I have lost friends/relatives after telling them that I was infected with COVD-19.	Agree	2	
		Disagree	0	
		Can't Say	1	
Q503	My family has gone through a lot of difficulties because of my COVID-19 status.	Agree	2	
		Disagree	0	
		Can't Say	1	
Q504	Some people avoid touching me even after my recovery once they know I was infected with COVD-19.	Agree	2	
		Disagree	0	
		Can't Say	1	
Q505	I have been insulted/discriminated because of my history of being infected with COVD-19.	Agree	2	
		Disagree	0	
		Can't Say	1	
Q506	The way people treat me makes me feel unwanted.	Agree	2	
		Disagree	0	
		Can't Say	1	
Q507	I was denied health care services when the doctors found	Agree	2	

ICMR-National Institute of Medical Statistics, Delhi

	out I was infected with for COVD-19.	Disagree	0
		Can't Say	1
Q508	I/my family members were denied essential services when the service providers found out I was infected with COVD-19.	Agree	2
		Disagree	0
		Can't Say	1
Q509	Telling others that I was infected with COVD-19 is risky.	Agree	2
		Disagree	0
		Can't Say	1
Q510	I work hard to keep my COVID-19 history as a secret.	Agree	2
		Disagree	0
		Can't Say	1
Q511	I have stopped socializing with some people because of their reactions towards me due to my COVID-19 status.	Agree	2
		Disagree	0
		Can't Say	1
Q512	I am very careful about who I tell that I was infected with COVD-19 as I worry that they will disclose it to others.	Agree	2
		Disagree	0
		Can't Say	1
Q513	People only refer to me by my COVID-19 status.	Agree	2
		Disagree	0
		Can't Say	1

SECTION 6. MITIGATION OF STIGMA

Q. No.	QUESTIONS AND FILTERS	CODING CATEGORIES	
Q601	According to you, what measures should be taken to mitigate the COVID-19 stigma?<br><br>**[MULTPLE RESPONSES]**	**Circle appropriate responses**	
		A	Never spread names or identity of those affected or under quarantine or their locality on the social media.
		B	Avoid spreading fear and panic.
		C	Should not target healthcare and sanitary workers or police
		D	Should not label any community or area for spread of COVID-19
		E	Avoid addressing those under treatment as COVID victims. Address them as ''people recovering from COVID''.
		F	Appreciate efforts of people providing essential services and be supportive towards them and their families
		G	Share only the authentic information available on the website of Ministry of Health and Family Welfare, Govt. of India or the World Health Organisation.
		H	Cross check any information related to CoVID-19 from reliable sources before forwarding any messages on social media.
		I	Share positive stories of those who have recovered from COVID-19.
		J	Other (please specify)

Thank you for your time. We will be in touch with you if we need any additional information. Our best wishes are with you.

ICMR-National Institute of Medical Statistics, Delhi

COMMUNITY RESPONDENTS-II

<u>Factors Related to Covid-19 Stigma: A Mixed-Methods Study</u>

SECTION 1: IDENTIFICATION

SECTION 2: SOCIOECONOMIC BACKGROUND

Q. No.	QUESTIONS AND FILTERS	CODING CATEGORIES	SKIP TO
Q201	Age (In completed years) 18-99	[ ][ ]	
Q202	Gender	Male 1<br>Female 2<br>Transgender 3	
Q203	Have you ever attended school	YES 1<br>NO 2	If 2 skip to Q205
Q204	What is the highest grade you completed?<br><br>***FOR CODE REFER MANUAL***	Grade 1 to 20 [ ]	If any grade 1 to 20 skip to Q206
Q205	If never attended school	Can read and write only 1<br>Can read only 2<br>Don't know 3	
Q206	What is your occupation?	Government Employee 1<br>Private Employee 2<br>Skilled Labour 3<br>Unskilled Labour 4<br>Business/Self Employee 5<br>Unemployment 6<br>Housewife 7<br>Student 8<br>Others (Specify) 9	
Q207	What is your family's monthly income (INR)?	Up to 5000 1<br>5001-7500 2<br>7501-10000 3<br>10001-20000 4<br>20001-50000 5<br>50001-100000 6<br>Above 100000 7	
Q208	What is your current marital status?	Never married 1<br>Currently married 2<br>Separated 3<br>Divorced/ separated 4<br>Widower/Widow 5<br>Live-in relationship 6	
Q209	Which religion do you belong to?	Hindu 1<br>Muslim 2<br>Christian 3<br>Budhist 4<br>Others (specify) 5	
Q210	Which caste/ tribe do you belong to?	Scheduled Caste 1<br>Scheduled Tribe 2<br>OBC 3<br>General 4<br>None of these 5	
Q211	Are you a usual resident of this place?	YES 1<br>NO 2	
Q212	How long have you been residing in	[ ][ ]	

ICMR-National Institute of Medical Statistics, Delhi

	this place? (Completed years)		
Q213	Whether you or any member of your family have visited any place out of your current place of residence since 1st March 2020?	YES 1<br>NO 2	
Q214	Whether you or any member of your family has returned to your current place of residence after 1st March 2020?	YES 1<br>NO 2	
Q215	Have you or your family (who are living with you currently) been quarantined or isolated for COVID-19?	YES 1<br>NO 2	If 2 skip to 301
Q216	If yes, could you tell us the reason	Family member positive 1<br>Neighbour positive 2<br>Co-worker positive 3<br>Others (Specify) 4	

SECTION 3: UNDERSTANDING OF COVID-19

Q. NO.	QUESTIONS AND FILTERS	CODING CATEGORIES	SKIP TO
Q301	Have you ever heard about COVID-19 or CORONA?	YES 1<br>NO 2	If 2 skip to Q304
Q302	Through which sources have you come to know about COVID-19?<br><br>**[MULTIPLE RESPONSES]**	**Circle appropriate responses**<br>A Newspaper<br>B Magazine<br>C Radio<br>D Television<br>E Internet<br>F Social platforms (E.g. WhatsApp, WeChat, Facebo Twitter, Instagram)<br>G Your doctor<br>H Your family or friends<br>I Other (please specify)	
Q303	Which one of the following sources of information do you trust the most?	Newspaper<br>Magazine<br>Radio<br>Television<br>Internet<br>Social platforms (E.g. WhatsApp, WeChat, Facebook, Twitter, Instagram)<br>Your doctor<br>Your family or friends<br>Other (please specify)	
Q304	COVID-19 is caused by:<br><br>**[MULTPLE RESPONSES]**	**Circle appropriate responses**<br>A A germ<br>B Virus<br>C By Bat<br>D Eating sea food or any animal food/ meat products<br>E Other (please specify)	
Q305	COVID-19 transmitted through:<br><br>**[MULTIPLE RESPONSES]**	**Circle appropriate responses**<br>A Air (when a person coughs, sneezes or talks)<br>B Touching another person<br>C Unknowingly touching a surface/object which has the	

ICMR-National Institute of Medical Statistics, Delhi

Q.No.	Question	Option	Response	Code
			virus and then touching your eyes and nose	
		D	Consumption of meat/chicken/sea food	
		E	Visiting butcher shops and fish markets	
		F	Caused by contaminated water	
		G	Other (please specify)	
Q306	Symptoms of COVID19 are:<br><br>**[MULTIPLE RESPONSES]**	**Circle appropriate responses**		
		A.	Fever	
		B.	Cough	
		C.	Difficulty breathing	
		D.	Nasal Congestion	
		E.	Running nose	
		F.	Sore Throat	
		G.	Loss of smell and Taste	
		H.	Fatigue	
		I.	Diarrhoea	
		J.	Constipation	
		K.	Rash	
		L.	Asymptomatic (No symptoms)	
		M.	Any Pain (head, body, muscle, chest, back)	
		N.	Other (please specify)	
Q307	It is possible that the COVID positive individual can be completely asymptomatic for some time		True	1
			False	2
			Don't know	3
Q308	COVID-19 can be prevented through:<br><br>**[MULTIPLE RESPONSES]**	**Circle appropriate responses**		
		A	Wearing a face mask	
		B	Washing hands frequently (With soap or hand saniti	
		C	Practising physical distancing	
		D	Avoiding contact with people who have sympto cough	
		E	Avoiding contact with people who have a travel his a foreign country	
		F	Avoiding crowded areas	
		G	Not visiting butcher shops and fish markets	
		H	Avoiding visits to a hospital or clinic	
		I	Avoiding public transportation	
		J	Being at home	
		K	Other (please specify)	
Q309	In your opinion, which of the 3 measures that people will adopt the most to prevent COVID transmission?	**Circle appropriate responses**		
		A	Wearing a face mask	
		B	Washing hands frequently (With soap or hand sanitizer)	
		C	Practising physical distancing	
		D	Avoiding contact with people who have symptoms of cough	
		E	Avoiding contact with people who have a travel history to a foreign country	
		F	Avoiding crowded areas	
		G	Not visiting butcher shops and fish markets	
		H	Avoiding visits to a hospital or clinic	
		I	Avoiding public transportation	
		J	Being at home	
		K	Other (please specify)	

ICMR- National Institute of Medical Statistics, Delhi

SECTION 4: RISK PERCEPTION

Q. No.	QUESTIONS AND FILTERS	CODING CATEGORIES		Skip to
Q401	According to you, who are at the risk of getting COVID-19? [MULTIPLE RESPONSES]	**Circle appropriate responses**		
		A	People who have a travel history from a foreign count	
		B	Young people	
		C	Children	
		D	Elderly	
		E	People living in crowded areas	
		F	People living in cities	
		G	People living in rural areas	
		H	Women	
		I	Men	
		J	People with any comorbidity	
		K	Other (please specify)	
Q402	According to you, who can spread COVID-19? [MULTPLE RESPONSES]	**Circle appropriate responses**		
		A	Doctors/health care workers	
		B	Police personnel	
		C	People who have a travel history from a foreign count	
		D	Young people	
		E	Children	
		F	Elderly	
		G	People living in crowded areas	
		H	People living in cities	
		I	People living in rural areas	
		J	Women	
		K	Men	
		L	People who do not practice social distancing	
		M	People who do not wear masks	
		N	Vendors (Vegetable, milk etc)	
		O	Other (please specify)	
Q403	On a scale of 1-5, how likely do you think you might become infected with COVID-19?	Very Unlikely	1	If 1 or 2 skip to Q405
		Unlikely	2	
		Neutral	3	
		Likely	4	
		Very likely	5	
Q404	Please elaborate the reasons why you think you might become infected with COVID-19? [MULTPLE RESPONSES]	**Circle approriate responses**		If A to M skip to Q 406
		A.	Come in close contact with COVID positive person	
		B.	Occupation	
		C.	Travel history from a foreign country	
		D.	Travel history from a place (in India) that has many COVID-19 people	
		E.	Elderly	
		F.	Prior illness (BP/Diabetes/TB/Cancer/ any other)	
		G.	Live in a crowded area	
		H.	Live in a place where infections are increasing	
		I.	Many people have come have come from cities to the rural area	
		J.	Pregnant	
		K.	Lactating mother	
		L.	Travel by public transportation	
		M	Any other response (please specify)	
Q405	Please elaborate the reasons why	**Circle appropriate responses**		

ICMR-National Institute of Medical Statistics, Delhi

	you think you might not become infected with COVID-19? **[MULTPLE RESPONSES]**	A.	Have not come in close contact with COVID positive person		
		B.	Always adhere to preventive measures		
		C.	Have no travel history from a foreign country		
		D.	Have no travel history from a place (in India) that has many COVID-19 people		
		E.	Young		
		F.	No prior illness		
		G.	Do not live in a crowded area		
		H.	Live in a place where there are no infections		
		I.	Do not take by public transportation		
		J.	Other (please specify)		
Q406	Did any of your family members who are staying with you test positive for COVID-19?		YES	1	If 1 skip to Q501
			NO	2	
Q407	How likely do you think your family members might become infected with COVID-19?		Very Unlikely	1	If 1 or 2 skip to Q409
			Unlikely	2	
			Neutral	3	
			Likely	4	
			Very likely	5	
Q408	Please elaborate the reasons why you think your family might become infected with COVID-19? **[MULTPLE RESPONSES]**		**Circle appropriate response**		If A to K then skip to Q 501
		A.	Have come in close contact with COVID positive person		
		B.	Occupation that requires close interaction with COVID-19 positive people		
		C.	Travel history from a foreign country		
		D.	Travel history from a place (in India) that has many COVID-19 people		
		E.	Are above 60 years of age		
		F.	Have prior illness (BP/Diabetes/TB/Cancer/ any other)		
		G.	Live in a crowded area		
		H.	Live in a place where infections are increasing		
		I.	Many people have come have come from cities to the rural areas where they stay		
		J.	Travel by public transportation		
		K.	Any other response (please specify)		
Q409	Please elaborate the reasons why you think your family might not become infected with COVID-19? **[MULTPLE RESPONSES]**		Circle appropriate responses		
		K.	Have not come in close contact with COVID positiv person		
		L.	Always adhere to preventive measures		
		M.	Have no travel history from a foreign country		
		N.	Have no travel history from a place (in India) that ha many COVID-19 people		
		O.	All are young		
		P.	Have no prior illness		
		Q.	Do not live in a crowded area		
		R.	Live in a place where there are no infections		
		S.	Do not take by public transportation		
		T.	Other (please specify)		

SECTION 5: STIGMA AGAINST COVID-19 POSITIVE PATIENTS IN THE COMMUNITY

READ THE FOLLOWING STATEMENTS CAREFULLY AND TICK ONE OPTION THAT APPLIES TO THE RESPONDENT.

We would like to understand your experiences with COVID-19. This understanding would help us provide information to help others diagnosed with COVID-19 to have better experiences as this is a new pandemic and there are still problems that need to be addressed. We will read you some statements and we would like you to respond if you agree/ disagree or if you cannot say. I will be happy to repeat the question if I am not clear

ICMR-National Institute of Medical Statistics, Delhi

Sl. No	STATEMENT	CODING CATEGORIES		
Q501	People infected with COVID-19 are always careless and spread the disease.	Agree<br>Disagree<br>Can't Say	2<br>0<br>1	
Q502	Most people are uncomfortable around COVID infected people even after their results are negative and after they are discharged from the hospital.	Agree<br>Disagree<br>Can't Say	2<br>0<br>1	
Q503	People with COVID-19 are treated as outcast.	Agree<br>Disagree<br>Can't Say	2<br>0<br>1	
Q504	If a person was infected with for COVID-19, it is better to avoid his/her family members.	Agree<br>Disagree<br>Can't Say	2<br>0<br>1	
Q505	People with the COVID-19 disease got what they deserve.	Agree<br>Disagree<br>Can't Say	2<br>0<br>1	
Q506	People infected with COVID-19 should continue to be isolated even after their recovery.	Agree<br>Disagree<br>Can't Say	2<br>0<br>1	

SECTION 6: MITIGATION OF STIGMA

Q. No.	QUESTIONS AND FILTERS	CODING CATEGORIES	
Q601	According to you, what should be done to mitigate the COVID-19 stigma?<br><br>**[MULTPLE RESPONSES]**	**Circle appropriate responses**	
		A	Never spread names or identity of those affected or under quarantine or their locality on the social media.
		B	Avoid spreading fear and panic.
		C	Should not target healthcare and sanitary workers or police
		D	Should not label any community or area for spread of COVID-19
		E	Avoid addressing those under treatment as COVID victims. Address them as ''people recovering from COVID''.
		F	Appreciate efforts of people providing essential services and be supportive towards them and their families
		G	Share only the authentic information available on the website of Ministry of Health and Family Welfare, Govt. of India or the World Health Organisation.
		H	Cross check any information related to CoVID-19 from reliable sources before forwarding any messages on social media.
		I	Share positive stories of those who have recovered from COVID-19.
		J	Other (please specify)

Thank you for your time. We will be in touch with you if we need any additional information. Our best wishes are with you.

ICMR-National Institute of Medical Statistics, Delhi
